# Combined use of veno‐venous extracorporeal membrane oxygenation and asynchronous independent lung ventilation after thoracic surgery for lung abscess

**DOI:** 10.1002/ccr3.8354

**Published:** 2023-12-28

**Authors:** Ryuhei Yoneda, Takeo Matsuyoshi, Tatsuro Yogi, Yuichi Sato, Jun Hamaguchi, Keiki Shimizu

**Affiliations:** ^1^ Department of Emergency Medicine Tokyo Metropolitan Tama Medical Center Tokyo Japan; ^2^ Department of Emergency Medicine Urasoe General Hospital Urasoe Japan

**Keywords:** extracorporeal membrane oxygenation, lung abscess, pneumothorax, pulmonary atelectasis, respiratory insufficiency

## Abstract

We used independent lung ventilation (ILV) during veno‐venous extracorporeal membrane oxygenation (V‐V ECMO) after lung abscess surgery in a patient with severe hypoxia and air leak. ILV can be effective in V‐V ECMO as unilateral lung air leak.

## Graphical abstract

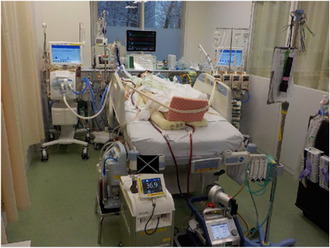

## 1 INTRODUCTION

Veno‐venous extra‐corporeal membrane oxygenation (V‐V ECMO) is used in perioperative respiratory care of patients with lung abscess who are considered unfit for one‐lung ventilation or who are functionally inoperable, despite exploiting all conventional mechanical ventilation possibilities, including nitric oxide inhalation. V‐V ECMO does not pose higher odds of postoperative mortality.^1^

Independent lung ventilation (ILV) is a mechanical ventilation treatment for differential lung disease, used in intensive care settings. ILV is divided into anatomical and physiological lung separation. Physiological lung separation is divided into synchronous and asynchronous ventilation.^2^

Combined use of V‐V ECMO and asynchronous ILV can have a perioperative advantage of lung abscess because in lung abscess, the right and left lungs often have different characteristics.

Herein, we describe the case of a patient who received V‐V ECMO for thoracic surgery for lung abscess, in whom ILV was performed to provide asynchronous treatment during the perioperative period.

## 2 CASE PRESENTATION

A 33‐year‐old man was admitted to a hospital after developing stomachache 2 weeks earlier, where he was diagnosed with an intestinal obstruction caused by ileocecal cancer. He had no relevant medical history. There, the ileocecal cancer was surgically removed, and the intestinal obstruction relieved. Thereafter, the patient aspirated, presented with respiratory failure, and required intubation. Upon intubation, the patient developed right pneumothorax, and developed a lung abscess after chest tube insertion. A day later, the patient presented with severe hypoxia and major right lung air leak was referred to our hospital for V‐V ECMO, primarily to address the severe air leak. We started V‐V ECMO at the initial hospital and then transferred him to our hospital (Day 1).

At V‐V ECMO initiation, the patient's Murray score was three points (ratio of partial pressure of carbon dioxide in arterial blood [PaO_2_]/fraction of inspired oxygen [FiO_2_], 107; positive end‐expiratory pressure [PEEP], 8 cmH_2_O; consolidation of all the lobes on chest X‐ray; pulmonary compliance, 18.8 mL/cmH_2_O). The first blood test date at our hospital are shown in Table 1. It showed increased levels of APTT, fibrinogen, fibrinogen/fibrin degradation products, D‐dimer, creatinine kinase, lactate dehydratase, C‐reactive protein, PaCO_2_, HCO_3_^−^, and decreased levels of hemoglobin, platelet count, total protein, and albumin.

**TABLE 1:** The first blood test date at our hospital.

Blood count, biochemistry, and coagulation date					
WBC	5.2 10^3^/μL	FDP	46.6 μg/mL	K	4.7 mmol/L
RBC	264 10^4^/μL	D‐D	22.0 μg/dL	Ca	6.7 mg/dL
HB	7.7 g/dL	TP	3.7 g/dL	CK	261 U/L
HCT	24.6%	ALB	0.9 g/dL	AST	19 U/L
PLT	65 10^3^/μL	BUN	9.7 mg/dL	ALT	37 U/L
PT(%)	105%	CRE	0.2 mg/dL	LDH	431 U/L
APTT	34.5 s	Na	150 mmol/L	AMY	21 U/L
FIB	459 mg/dL	Cl	112 mmol/L	CRP	25.8 mg/dL
Arterial blood gas					
PH	7.38				
PaO_2_	71.4 mmHg				
PaCO_2_	57.3 mmHg				
HCO_3_^−^	33.1 mmol/L				
Lactate	1.4 mmol/L				
Glucose	179 mg/dL				

Abbreviations: ALB, albumin; ALT, alanine aminotransferase; AMY, amylase; APTT, activated partial thromboplastin time; AST, aspartate aminotransferase; BUN, blood urea nitrogen; CK, creatine kinase; CRE, creatinine; CRP, C‐reactive protein; D‐D, D‐dimer; FDP, fibrinogen/fibrin degradation products; FIB, fibrinogen; HB, hemoglobin; HCT, hematocrit; LDH, lactate dehydrogenase; PLT, platelet; PT, prothrombin time; RBC, red blood cell; TP, total protein; WBC, white blood cell.

After introducing V‐V ECMO, we changed the ventilator settings to the lung rest settings (FiO_2,_ 0.4; ventilation rate [f], 6; PEEP, 10 cmH_2_O; inspiratory pressure [IP], 10 cmH_2_O). Nevertheless, right lung air leakage continued. We then changed the intubation tube to a double lumen tube (DLT) and clamped the right‐side lumen. On Day 4, enhanced computed tomography (CT) indicated thoracic surgery for lung abscess drainage and air leak closure.

**FIGURE 1:**
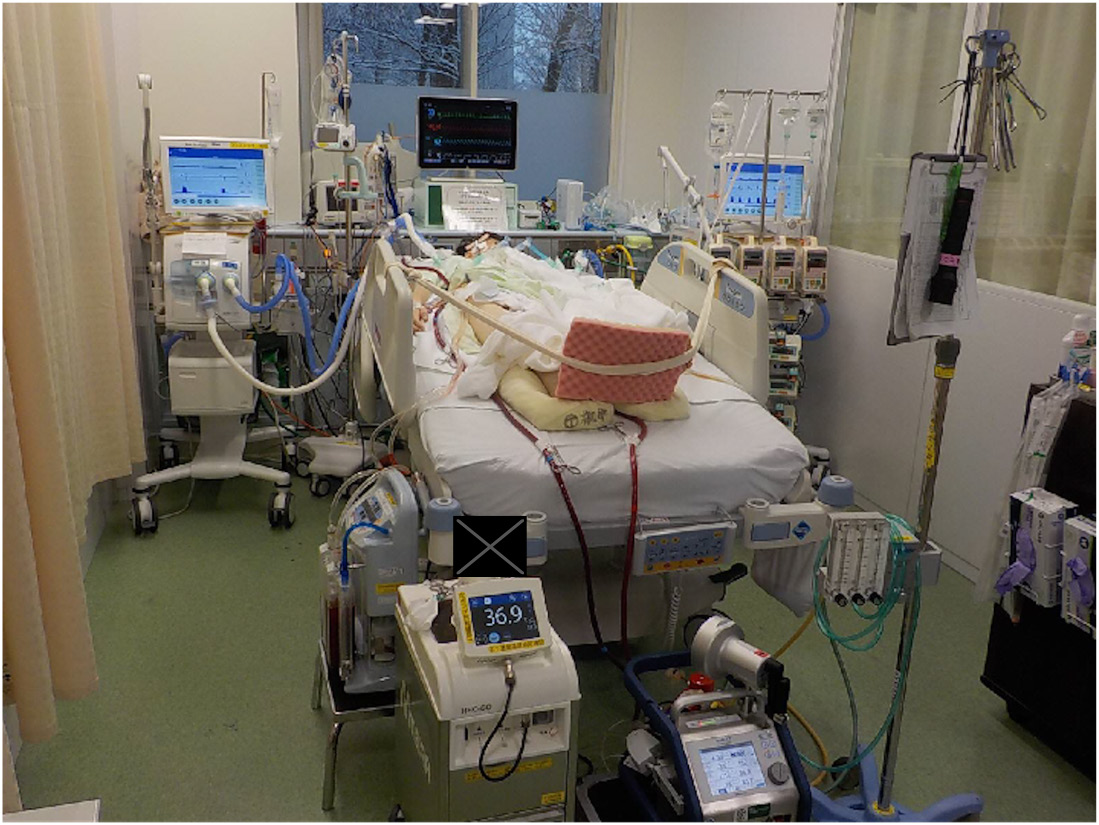
After thoracic surgery, independent lung ventilation (ILV), which aimed at asynchronous treatment with physiological lung separation, was started. Right ventilator settings: FiO_2,_ 0.4; f, 10; positive end‐expiratory pressure (PEEP), 5 cmH_2_O; inspiratory pressure, 5 cmH_2_O. Left ventilator settings: FiO_2_, 0.4; f, 16; PEEP, 12 cmH_2_O; IP, 10 cmH_2_O.

On Day 5, right lower lung lobectomy, median lobe segmentectomy, and thoracoplasty were performed. ILV was then started for physiological lung separation (Figure 1).

On Day 6, we performed a tracheostomy and changed the DLT to tracheostomy DLT. The right lung ventilator settings gradually approached those of the left ventilator. On Day 12, ILV ended because the ventilator settings of the right lung reached those of the left lung, and we changed a tracheostomy DLT to the tracheostomy tube.

On Day 17, we performed enhanced CT again because the patient was hemodynamically unstable and we suspected sepsis, indicating right lung abscess aggravation. Re‐thoracotomy was performed, and we initiated chest cavity lavage every few days.

On Day 23, air leakage relapsed. We changed the tracheostomy tube for a tracheostomy DLT to stop right lung ventilation. On Day 25, air leaked in the right lung during washing of the chest cavity; the leak was closed.

On Day 32, we again started ILV, which ended on Day 38, without air leakage relapse.

**FIGURE 2:**
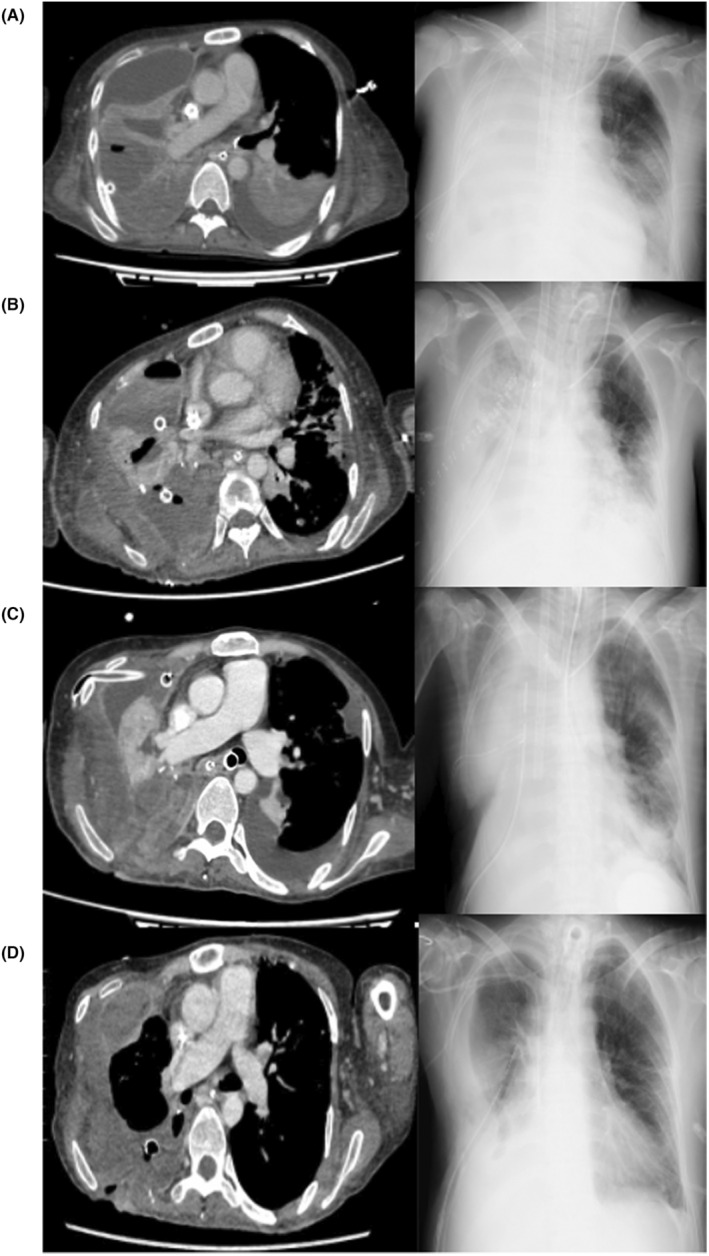
The transition of chest computed tomography (CT) and chest X‐ray. (A) Day 4. We confirmed the characteristics of the lung abscess. As intercurrent air leakage was present, we decided to perform thoracic surgery. (B) Day 17/18. Chest CT was performed on Day 17, and chest X‐ray on Day 18. On Day 17, we performed CT because the circulatory dynamics were unstable and we suspected sepsis. On the same day, re‐thoracotomy was performed. (C) Day 27. The right lung was pale in appearance. On Day 32, independent lung ventilation (ILV) was again started for the purpose of physiological lung separation. After that, transparency of the right lung gradually improved. (D) Day 56. Veno‐venous extracorporeal membrane oxygenation was decannulated on Day 48. Transparency of the right lung improved.

We successfully performed a gas clamp test of V‐V ECMO on Day 46. V‐V ECMO was decannulated on Day 48. The patient's thoracic cavity was closed on Day 49, after which tidal volume increased. Table 2 describes changes in tidal volume and ventilator settings from Day 25 to 55. Figure 2 shows the progression of chest CT and chest X‐ray. The patient was transferred back to the initial hospital on Day 61.

**TABLE 2:** Changes in tidal volume and ventilator settings.

Day	ECMO			Right lung			Left lung			Total lung		
	Flow	Sweep gas	FiO_2_	TV	PEEP	IP	TV	PEEP	IP	TV	PEEP	IP
25	4.59	4	1	0			70	13	25			
26	4.17	4	1	0			91	13	12			
27	4.24	4	1	0			81	13	12			
28	4.15	4	1	0			116	8	19			
29	3.06	3	1	0			89	10	15			
30	3.11	2	0.6	0			80	12	15			
31	3.33	4	0.8	0			115	12	15			
32	3.24	3	0.6	5	5	5	93					
33	3.16	2	0.6	6	5	5	80					
34	3.09	1	0.6	5	7	7	93					
35	3.53	0.5	0.6	7	7	7	133					
36	3.55	0.5	0.6	7	7	7	158					
37	3.95	0.5	0.6	7	7	7	121					
38	4.08	0.5	0.6							168	12	15
39	4.12	0.5	0.4							159	12	15
40	4.12	0.5	0.4							168	9	15
41	4.28	0.5	0.4							184	9	15
42	4.44	0.5	0.4							162	9	15
43	4.35	0.5	0.4							194	9	15
44	4.41	0.5	0.4							155	9	15
45	3.88	0.5	0.4							187	9	15
46	4	Clamp	Clamp							211	8	15
47	4.05									210	8	15
48										184	8	15
49										230	8	15
50										202	9	15
51										200	9	15
52										235	9	15
53										269	7	15
54										212	7	15
55										277	7	15

*Note*: Tidal volume (TV) and ventilator settings from Day 25 to 55. On Day 32, we again started independent lung ventilation (ILV) with physiological lung separation. ILV in this phase ended on Day 38. Gas weaning of veno‐venous extracorporeal membrane oxygenation (VV‐ECMO) was started on Day 29. We performed a gas clamp test of VV‐ECMO from Day 46 to 48, and VV‐ECMO was decannulated on Day 48. We closed the patient's thoracic cavity on Day 49. The TV increased after closing the thoracic cavity.

Abbreviations: IP, inspiratory pressure; PEEP, positive end‐expiratory pressure.

## 3 DISCUSSION

Lung abscesses are classified as primary or secondary, depending on whether they occur in the absence or presence of underlying pulmonary lesions.^3^ They are also divided into acute (<6 weeks) and chronic (>6 weeks). Antibiotic therapy, thoracic cavity drainage, and surgical resection are used as therapy. Acute indications for surgical resection include hemoptysis, prolonged sepsis and febricity, bronchopleural fistula, and abscess rupture in the pleural cavity with pyopneumothorax/empyema,^4^ as in our case.

A previous report described the use of V‐V ECMO for perioperative management of 10 lung abscess cases, of whom nine survived.^1^ Our patient underwent both thoracic surgery for lung abscess and asynchronous ILV during V‐V ECMO. In anatomical ILV, one lung is separated from the other, typically as a short‐term rescue treatment strategy for massive hemoptysis and inter‐bronchial secretion aspiration in a critical care setting. Physiological lung separation is applied when lungs have different characteristics, due to unilateral lung disease or injury, thus allowing treatment strategies to each lung. Physiological lung separation can be synchronous or asynchronous treatment. In synchronous treatment, the respiration frequency is the same for both lungs, while other ventilatory parameters (tidal volume, IP, PEEP, and FiO_2_) are individually set for each lung. In asynchronous treatment, the ventilation mode and all ventilatory parameters including respiratory rate for each lung can differ. Despite initial concerns that asynchronous ILV may cause cardiovascular compromise due to decreased venous return, as inflation of each lung at different times would result in elevated intrathoracic pressure for a longer period, it is well tolerated. Asynchronous ventilation using two ventilators is simpler, offers greater scope for individual titration of ventilator parameters, and is thus preferred for ILV.^2^, ^5^

Some previous reports described use of V‐V ECMO with ILV.^6^, ^7^ By entrusting primary gas exchange to V‐V ECMO, ventilator settings during V‐V ECMO aim to avoid ventilator‐induced lung injury. The Extracorporeal Life Support Organization guidelines recommend PEEP ≥10 mmH_2_O to avoid atelectasis.^8^

We opted for asynchronous ILV with physiological lung separation, because the left lung was healthy and did not require a low respiratory rate and PEEP. Otherwise, ventilator settings considering right lung leakage could have caused left lung atelectasis. Second, the ventilator settings for the right lung gradually approached those of the left lung postoperatively. While we considered the right lung air leak, atelectasis in the right lung should be avoided, given the possibility of right lung recovery. The patient's tidal volume increased after thoracic cavity closure, possibly partially due to right lung recovery, made possible by asynchronous treatment with physiological lung separation.

## 4 CONCLUSION

Asynchronous ILV treatment with physiological lung separation is effective during V‐V ECMO. This combination can realize optimal ventilator settings for the right and left lungs when they have different characteristics in V‐V ECMO management, such as unilateral air leakage.

## AUTHOR CONTRIBUTIONS

**Ryuhei Yoneda:** Writing – original draft. **Takeo Matsuyoshi:** Writing – review and editing. **Tatsuro Yogi:** Resources. **Yuichi Sato:** Data curation; resources. **Jun Hamaguchi:** Data curation; resources; supervision. **Keiki Shimizu:** Project administration.

## CONFLICT OF INTEREST STATEMENT

None declared.

## CONSENT

Written informed consent was obtained from the patient to publish this report.

## Data Availability

All data available has been presented within this manuscript.

## References

[1] Schweigert M , Dubecz A , Giraldo Ospina CF , et al. Use of extracorporeal membrane oxygenation in non‐elective major thoracic surgery for infectious lung abscess. Eur J Cardiothorac Surg. 2022;62(4):ezac116. doi:10.1093/ejcts/ezac116 35213707

[2] Tehrani FT . Computerised decision support for differential lung ventilation. Healthc Technol Lett. 2019;6(2):37‐41. doi:10.1049/htl.2018.5091 32082591 PMC7010243

[3] Sabbula BR , Rammohan G . Lung Abscess AJ. StatPearls. StatPearls Publishing; 2022. 32310380

[4] Kuhajda I , Zarogoulidis K , Tsirgogianni K , et al. Lung abscess‐etiology, diagnostic and treatment options. Ann Transl Med. 2015;3(13):183. doi:10.3978/j.issn.2305-5839.2015.07.08 26366400 PMC4543327

[5] Berg S , Bittner EA , Berra L , Kacmarek RM , Sonny A . Independent lung ventilation: implementation strategies and review of literature. World J Crit Care Med. 2019;8(4):49‐58. doi:10.5492/wjccm.v8.i4.49 31667133 PMC6817931

[6] Shin K , Hifumi T , Tsugitomi R , et al. Empyema with fistula successfully treated with a comprehensive approach including bronchial blocker and embolization receiving veno‐venous extracorporeal membrane oxygenation. Acute Med Surg. 2021;8(1):e621. doi:10.1002/ams2.621 33604054 PMC7871201

[7] Bhargava V , Arastu A , Darling C , Wang E , Kache S . Differential lung ventilation using a bronchial blocker in a pediatric patient on extracorporeal membrane oxygenation: a case report. A A Pract. 2019;13(6):206‐210. doi:10.1213/XAA.0000000000001025 31162224

[8] Tonna JE , Abrams D , Brodie D , et al. Management of adult patients supported with venovenous extracorporeal membrane oxygenation (VV ECMO): guideline from the extracorporeal life support organization (ELSO). ASAIO J. 2021;67(6):601‐610. doi:10.1097/MAT.0000000000001432 33965970 PMC8315725

